# Latest advances in biomimetic nanomaterials for diagnosis and treatment of cardiovascular disease

**DOI:** 10.3389/fcvm.2022.1037741

**Published:** 2023-01-04

**Authors:** Yuxuan Gong, Huaying Liu, Shen Ke, Li Zhuo, Haibin Wang

**Affiliations:** ^1^College of Life Sciences and Bioengineering, School of Physical Science and Engineering, Beijing Jiaotong University, Beijing, China; ^2^Department of Nephrology, China-Japan Friendship Hospital, Beijing, China

**Keywords:** cardiovascular disease, nanomedicine, nanomaterials, theranostics, heart failure

## Abstract

Cardiovascular disease remains one of the leading causes of death in China, with increasingly serious negative effects on people and society. Despite significant advances in preventing and treating cardiovascular diseases, such as atrial fibrillation/flutter and heart failure over the last few years, much more remains to be done. Therefore, developing innovative methods for identifying and managing cardiovascular disorders is critical. Nanomaterials provide multiple benefits in biomedicine, primarily better catalytic activity, drug loading, targeting, and imaging. Biomimetic materials and nanoparticles are specially combined to synthesize biomimetic nanoparticles that successfully reduce the nanoparticles’ toxicity and immunogenicity while enhancing histocompatibility. Additionally, the biological targeting capability of nanoparticles facilitates the diagnosis and therapy of cardiovascular disease. Nowadays, nanomedicine still faces numerous challenges, which necessitates creating nanoparticles that are highly selective, toxic-free, and better clinically applicable. This study reviews the scientific accomplishments in this field over the past few years covering the classification, applications, and prospects of noble metal biomimetic nanozymes and biomimetic nanocarriers.

## 1. Introduction

Nanomaterials refer to materials with a single unit size of between 1 and 1,000 nanometers, having been widely studied and applied in many fields (1). Nanomaterials exhibit the major advantages of better drug loading, targeting, and imaging in biomedicine. However, disadvantages such as partial cytotoxicity and immunogenicity displayed by various nanomaterials have wider clinical applications (2, 3).

The term “nanomedicine” introduced by Drexler et al. has been used to describe any nanomaterial for diagnostic or therapeutic applications. Nanoparticles, the most common form of nanomedicines, are classified according to their composition into lipid nanoparticles, protein nanoparticles, inorganic/metallic nanoparticles, and drug nanocrystals. Based on their basic properties, nanoparticles form a class of materials with unique physical and chemical properties finding a wide range of applications (4).

Biomimetic materials and nanomaterials are the two components that make up biomimetic nanomaterials. Biomimetic nanoparticles with specific biological features are synthesized by carefully combining nanoparticles with biomimetic materials (such as cell membranes, lipoproteins, viruses, etc.). This way, the original nanoparticles’ toxicity and immunogenicity are successfully decreased. However, the nanoparticles should also have better histocompatibility and biological targeting, thus allowing for improved disease detection and treatment (5–7).

Inflammation, the most frequent pathogenic process in the human body, is the immune system’s protective response to damage-induced agents in body tissue (8). Inflammation-associated disorders are categorized into acute and chronic inflammatory types, where trauma, infection, and acute myocardial infarction are examples of the former. In contrast, atherosclerosis, arthritis, and tumor immunity belong to the latter (9, 10).

Despite significant advances in the prevention and treatment of cardiovascular disease over the last few years, much more remains to be done. Therefore, it is critical to developing innovative methods for identifying and managing cardiovascular disorders (11, 12). Biomimetic nanoparticles are a class of potential drugs for treating cardiovascular disease. Nanomedicine-based drug delivery systems should possess superior physical and chemical properties, including tiny size, good selectivity, and high tissue permeability. Consequently, biomimetic nanomaterials have increasingly gained popularity among researchers in recent years (13). This review focuses on the latest developments of biomimetic nanomaterials and how they can be applied to the diagnosis and treatment of cardiovascular disease, particularly the repair of heart damage caused by AMI.

## 2. Noble metal biomimetic nanozymes

Nanozymes, a recently emerging synthetic enzyme that combines the properties of nanomaterials and enzymes, represent a promising alternative to naturally occurring enzymes. Noble metal-based nanozymes, a crucial part of nanozyme materials, have attracted great attention owing to their various advantages.

### 2.1. Development of noble metal-based nanozymes

“Nanozymes” are nanomaterials with enzymatic catalytic activity, which display efficient enzyme-like catalytic activity and the characteristics of nanomaterials. In addition, nanozymes have incomparable advantages over natural enzymes, such as low-cost preparation, high stability, and mass production. Nanomaterials have long been considered a class of chemically inert substances with no biological effects. Gao et al. (14) researchers discovered that magnetite (Fe_3_O_4_) nanoparticles demonstrate horseradish peroxidase (HRP)-like activity. Since then, many laboratories worldwide have also successfully reported on some nanomaterials with the properties of enzymes (15–17).

Nanozymes, a class of nanomaterials with enzymatic catalytic properties, open more prospects for treating cardiovascular diseases (18). In the past few years, the rapid advances in nanotechnology, bioengineering, enzymatic, and structure have been encouraging researchers to simulate new enzymatic activities and modulate nanoenzyme activities by preparing high-performance nanomaterials (19). Significant progress has also been achieved in elucidating catalytic mechanisms and exploring potential application areas. So far, more than 200 laboratories worldwide have explored nanozymes, and the research of nanozymes has been expanded into many fields, gradually spawning a new field of study (20). Compared with natural enzymes, the various advantages of nanozymes potentiate a wider range of applications. As an essential part of nanoenzyme materials, noble metal-based nanozymes have attracted much attention due to their high biocompatibility, simple synthesis, and easy modification (21). They have been studied and applied in multiple fields for bioactive molecular detection, disease diagnosis, treatment, antibacterial, etc (22).

**FIGURE 1:**
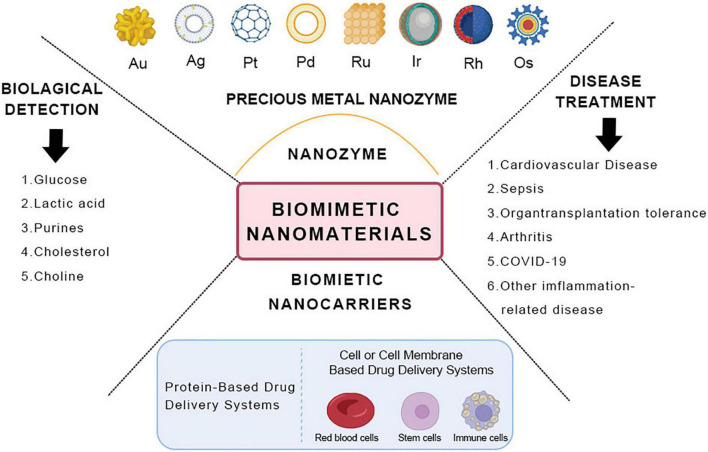
Schematic diagram of the classification and application of biomimetic nanomaterials.

Noble metal-based nanoparticles mainly include gold (Au), silver (Ag), platinum group metals—platinum (Pt), palladium (Pd), ruthenium (Ru), iridium (Ir), rhodium (Rh), osmium (Os), eight rare metal elements, etc (23). A noble metal is a vital catalyst material with superb optical, electrical, thermal, magnetic, and catalytic properties of the surface. It has favorable adsorption of reactants at moderate strength, contributing to the formation of intermediate “active compounds” (24). The most common uses for noble metals include medical treatment, biosensing, and catalysis (25, 26) (Figure 1).

### 2.2. Classification of noble metal-based nanozymes

Generally, enzymes can be classified according to their mechanisms: oxidoreductases, transferases, hydrolases, lyases, isomerases, ligases, and translocases. Most of the reported nanozymes can mimic the activities of oxidoreductases such as peroxidase (POD), oxidase (OXD), catalase (CAT), and superoxide dismutase (SOD); some nanozymes show a catalytic ability similar to hydrolases or the others.

Noble metals (such as Au, Ag, Pd, and Pt) exhibit varying enzyme-like activities under different conditions (27), with noble metal materials utilizing the uncoordinated atoms on the surface as the enzymatic reaction center. Under different conditions, noble metals can exhibit various enzymatic activities similar to OXD, POD, CAT, and SOD (28).

A small reserve of noble metals on the earth limits their applications as essential catalyst materials. To improve the utilization rate of noble metals and reduce their costs, the syntheses of non-noble metals with noble metals and non-noble metals with non-noble metals have been developed using doping methods (29, 30). Compared with single metals, bimetallic nanomaterials possess unique electronic structures and more favorable properties due to the synergistic effect of the two metals. The application of nanoalloys can reduce the usage of noble metals and enhance the catalytic activity of created nanomaterials (31). In recent years, the design and synthesis of bimetallic materials based on the elemental composition, microstructure, and other factors have been instrumental in substituting for noble metals and enhancing catalytic activity and selectivity (32).

The preparation of ultra-small nanozymes with simple structures and multi-enzyme-mimicking properties holds great potential for their applications in ROS scavenging (33–35). Meanwhile, the inherent ultra-small property of noble metal-based nanozymes is greatly helpful to alleviate the organism’s toxicity (36).

#### 2.2.1. Peroxidase mimetic activity

Peroxidases (PODs) typically catalyze substrates to oxidate when H_2_O_2_ or organic peroxides are consumed. Most natural peroxidases are heme proteins that activate H_2_O_2_ and generate high-valent intermediate species capable of extracting electrons from different substrates (37). The abundance of iron in heme proteins has contributed to the finding of many POD-mimicking iron-based nanomaterials (38, 39).

Innovative work by Gao et al. (14) showed that Fe_3_O_4_ nanoparticles intrinsically catalyze classical POD substrates, including 3,3,5,5-tetramethylbenzidine (TMB), Diaminobenzidine (DAB), and o-phenylenediamine (OPD) (40). Similar to these nanoparticles, other nanomaterials containing iron can also be used as POD mimetics. In addition, nanomaterials containing other transition metals, including Au (41), Ag (42), Pt (43), Pd (44), Ir (45), and Ru (46), can also serve as PODs (47).

Nanozyme studies on gold nanostructures or their mixtures with other metals are popular as they are easy to synthesize and modify on the surface, highly stable, and excellent in peroxidase activity (48). Many studies followed the first report of peroxidase activity in gold nanoparticles in 2004 to evaluate the peroxidase activity when the morphology was changed and they were hybridized with different metals (49).

#### 2.2.2. Oxidase mimetic activity

OXD usually participates in redox reactions at the expense of the molecular oxygen (O_2_), which is reduced to H_2_O_2_ or H_2_O. Comotti et al. reported that small-sized gold nanoparticles (AuNPs) could promote the glucose to converse into the gluconate, which behaves similarly, to glucose oxidase (GOx) (50). Since then, ultra-small nanomaterials based on noble metals, such as Au (51, 52), Ag (53), Pt (54), and Ir (55) have been demonstrated to be capable of OXD simulation. Gold nanoparticles (AuNPs) are effective nanomaterial-based enzyme mimics (nanozymes) for enzymatic reactions under mild conditions. AuNPs are one of the most widely studied nanoparticles and are highly inert nanomaterials (56). Recently, they presented surprisingly enzyme-mimicking properties under different surface modifications. Since the catalytic activity of NPs depends on their shape, size, and surface modification, the correlation between the structure and the performance contributes to the design of more efficient catalytic systems (57).

#### 2.2.3. Catalase mimetic activity

Catalase is a typical biocatalyst in almost all living systems that encourages H_2_O_2_ to break down into H_2_O and O_2_. Up to now, several noble metal nanoparticles have been demonstrated to have catalytic activity (58). Fan et al. (59) successfully synthesized highly stable platinum nanoparticles (Pt-Ft) sized between 1 and 2 nm using apoFt as the nucleation substrate. Pt-Ft was demonstrated to exhibit both catalytic and peroxidase activities by directly measuring the product of its catalytic reactions. With hydrogen peroxide as a substrate, oxygen bubbles were observed after the decomposition of hydrogen peroxide by Pt-Ft. Positively charged gold nanoparticles that possess intrinsic peroxidase-like activity can catalyze the oxidation of the peroxidase substrate 3,3,5,5-tetramethylbenzidine (TMB) by H_2_O_2_ to produce a blue color in aqueous solution, thus working as a simple method for the colorimetric detection of H_2_O_2_ and glucose (60).

#### 2.2.4. Superoxide dismutase mimetic activity

The superoxide radical (O^2–^) breakdown into O_2_ and H_2_O_2_ is catalyzed by SOD, which serves as a key antioxidant for the organism to defend against oxidative stress. Superoxide dismutase (SOD) is a crucial and naturally occurring enzyme for cells to defend against oxidation. It can catalyze the superoxide to break down, producing oxygen and hydrogen oxide. SOD-like activities can be seen in variable degrees in nanomaterials made of gold (61), platinum (62), palladium (63), ruthenium (64), and other noble metals. Wu et al. found that palladium nanoparticles could scavenge superoxide and display SOD-like activity, making them potential antioxidant defense in biological systems (65). Pt NPs with SOD-like activity was discovered by Miyamoto et al. and employed in antioxidant therapy in *Caenorhabditis*. *elegans.* The findings demonstrated that Pt NPs might prolong the life of *C. elegans* as they scavenged paraquat-induced H_2_O_2_ and superoxide anion O_2_ (66). The excellent SOD-like enzyme activity of Noble metal-based nanozymes has great potential in the application of antioxidant therapy.

#### 2.2.5. Ultra-small noble metal-based nanozymes

The size and morphology of nanozymes play a crucial role in determining enzyme-like activity because they are closely related to the specific surface area of nanozymes. Exposing more active sites due to their higher surface-area-to-volume ratio, smaller-sized nanomaterials have been shown to have better catalytic activity by numerous studies. Noble metal nanomaterials have such potential properties as easy synthesis, ultra-small size, easy surface modification, and specific physicochemical properties (67). Smaller nanozymes, however, can occasionally lose their enzymatic activity. In contrast to 2.9 nm Pt covered by cytosine-rich oligonucleotides, 1.8 nm Pt nanozymes covered by guanine-rich oligonucleotides had weaker peroxidase-like activity. The cause is that 2.9 nm Pt contains more metallic Pt^0^ than 1.8 nm Pt (68). In conclusion, the catalytic activity of ultra-small noble metal-based nanozymes is related to the surface area and metal valence.

#### 2.2.6. Bimetallic biomimetic nanozymes

The activity of peroxidase nanozymes based on AgM bimetallic alloys can be fine-tuned by gradually changing the ratio of the two metals (69). This is due to alloying, an enzymatic activity dependent on the compositions as the electronic structure changes. Growing with active nanomaterials or doping with another element is a cost-effective way to tune the activity (70).

Growing with less active nanomaterials such as Au and Ag together as well as with more active ones such as Pt and Ir can enhance the activity of enzymes but also effectively utilize these noble metals (71). Its catalytic effectiveness will be at least 20 times greater than that of Pd cubes and 400 times greater than that of HRP, respectively, after multiple atomic Ir layers are coated on Pd cubes (72).

One of the challenges in nanozyme-based nanotechnology is to be multifunctional in one material. Wu’s group synthesized a high-efficiency Au@Pt multifunctional nanozyme to detect H_2_O_2_ based on a seed-mediated method. This structure possesses both plasmonic activities from the Au core and enzymatic activity from the Pt shell, thus shortening the detection time and increasing the sensitivity by 1–2 orders of magnitude (73).

To further promote the activity, the less active core is selectively etched after the highly active core grows. For example, after the Pd core is etched, the Pd-Pt core-framework nanodendrites are transferred into the Pt hollow nanodendrites, accompanied by more active sites. Besides, high-refractive-index facets are exposed to enhance peroxidase-like activity (74).

Doping is another effective strategy to modulate enzyme activities benefiting from electronic structural changes. It has been demonstrated through PdO doping that creating heterojunctions on Co_3_O_4_ nanoparticles could quantitatively tune the band gap and Fermi level to investigate how metal oxide nanoparticles’ semiconductor properties affect cellular redox homeostasis and potential hazards (75).

### 2.3. Application

After explorations by researchers, the enzyme-like activity of noble metal-based nanozyme materials has been continuously improved. In some aspects, it has been able to replace biological enzymes for practical purposes (Table 1).

**TABLE 1:** The enzymatic mimetic activities of different precious metals and their specific therapeutic applications in several major ROS-related diseases and cardiovascular diseases.

Noble metal nanoparticles	Enzymatic activity	ROS-related diseases	Cardiovascular disease	References
Au	POD, OXD, CAT, SOD	Cancer	MI	(41, 51, 52, 58, 102, 116)
Ag	POD, OXD, CAT, SOD			(42, 184)
Pt	POD, OXD, CAT, SOD	Neurological disease, Skin diseases, Cancer		(43, 54, 66, 185)
Pd	POD, OXD, CAT, SOD	Skin diseases, Cancer		(44, 65, 100)
Ru	SOD	Cancer		(46, 186)
Ir	POD, OXD	Cancer, AKI		(45, 55)
Rh				(187)

It is well known that reactive oxygen species (ROS), such as superoxide anion (O^2–^), hydroxyl radical (⋅OH), and hydrogen peroxide (H_2_O_2_) are by-products of cellular metabolism (76, 77). Under normal circumstances, producing and removing ROS in the body are balanced dynamically. Low doses of ROS play an essential role in signal transduction, cell proliferation, and defense against pathogen invasion (78, 79). However, abnormally high ROS causes oxidative stress and disturbs the body’s redox equilibrium, significantly impairing the structure and function of the intracellular macromolecule (80, 81). Moreover, studies have shown that neurodegenerative diseases, cancers, diabetes, atherosclerosis, arthritis, and kidney diseases are all related to ROS accumulation (82–85). Given the anti-oxidative and pro-oxidative activities of nano-enzymes and their unique advantages, constructing nano-enzyme-based therapeutic systems and applying them in biomedicine, such as anti-inflammatory, cancer, and especially cardiovascular diseases, are discussed in the article.

#### 2.3.1. Biological detection

The HRP, as a natural enzyme, is often used to detect the content of H_2_O_2_. Nanozymes with peroxidase-like activity can substitute HRP. Studies have proved that nanozymes are more efficient and stable in detecting H_2_O_2_ (86, 87). The content of substrates can be reflected by that of H_2_O_2_, generated by the reaction between the substrates and corresponding enzymes. So far, glucose, lactic acid (88), choline (89), cholesterol (90), and other substances have been detected precisely. Gold nanoparticles have been on the cutting edge of sensing and detection technologies for simple synthesis, surface functionalization, and superior optical and electronic properties (91). Huang’s group directly grew AuNPs on two-dimensional MOF nanosheets to prepare AuNPs/Cu-TCPP (M) (TCPP = tetrakis (4-carboxyphenyl) porphyrin. *M* = Fe, Co) composite was used to detect glucose based on the chromogenic method (92). To better understand the enzymatic activities of AuNPs, Zhao et al. (93) prepared a three-in-one functional nanoplatform (sensing, self-assembly, and cascade catalysis). They found that glucose could be detected when only AuNPs were used.

Wu et al. (94) achieved strong electrochemiluminescence (ECL) emission by using one type of the G-quadruplex selective iridium (III) complex as an efficient ECL signal probe. Based on the typical sandwich immunoreaction between the cardiac troponin-I antigen (cTnI) and its corresponding antibody, the iridium (III) complex was intr.oduced according to its specific interaction with G-quadruplex DNA to modify on the surface of negatively charged gold nanoparticles (AuNPs). This induced a strengthened increased ECL signal proportional to cTnI concentration. Based on this, cTnI could be detected quantitatively ranging from 5.0 fg/mL to 100 ng/mL, with a detection limit of 1.67 fg/mL. Moreover, the proposed immunosensor successfully detected cTnI between healthy individuals and AMI patients in human serums. This suggested it was a potential candidate to be applied in the early diagnosis of AMI.

#### 2.3.2. Disease treatment

Similar to the application mechanism of nanozymes in the field of antibacterial, how to regulate the ROS level in organisms can also be used to treat diseases. Evidence suggests that ROS levels are closely related to the aging of the skin and other organs. Multiple ROS-resistant and repair systems of complex organisms can mitigate oxidative damage to tissues. Superoxide free radicals in living organisms have solid oxidizing properties, and their excessive levels can damage various tissues, causing diseases. Superoxide dismutase (SOD) is crucial to the antioxidant system. It can decompose superoxide radicals into H_2_O_2_, which is further decomposed into H_2_O and O_2_ after being catalyzed (95).

##### 2.3.2.1. Treatment of ROS-related diseases

Noble metal-based nanozymes can scavenge ROS and be used to treat ROS-related diseases, such as in antioxidant therapy (96, 97). In recent investigations, biorthogonal enzymes have been utilized to replace ROS to transform pharmacological precursors into hazardous medicines to treat cancer. These techniques have explored new ways to treat diseases with nanozymes (95).

The POD-like activity, photosensitization properties, and localized photothermal effect of noble metal-based nanozymes can be used to alleviate hypoxia at tumor sites and kill tumor cells photothermally and photodynamically (98–101). Therefore, noble metal-based nanozymes are a class of potential anti-tumor therapeutic materials. Gao et al. encapsulated polylactic acid with doxorubicin (DOX). Gold nanoparticles were grown on the surface of glycolic acid copolymer (PLGA). Then the surface was PEGylated to prepare multifunctional hybrid nanostructures with drug loading, CAT-like activity, photoacoustic imaging, and photothermal effect. The photothermal conversion efficiency of the obtained PLGA/DOX@PDA-Au-PEG hybrid structure was as high as 69.0% and a strong photoacoustic signal was produced. Under an 808 nm laser irradiation, the hybrid nanostructures enhanced ROS production, effectively alleviating the hypoxic condition at the tumor site. The release of DOX and the increase of ROS concentration enhanced the effect of chemotherapy/photothermal combined therapy on tumors (102).

##### 2.3.2.2. Cardiovascular disease treatment

Cardiovascular disease (CVD) is the leading cause of death worldwide, with a poor prognosis. Nanoscience involves the cross-integration of different sciences, including physics, materials science, engineering, and biomedicine; it sets the path to transform nanotechnology into clinical research. Given the unique physical, chemical, and biological properties of nanomaterials and nanostructures combined with the recent development of biomedical devices, contrast agents, and so forth, nanotechnology has great potential in cardiovascular regenerative medicine.

###### 2.3.2.2.1. Diagnosis and treatment of coronary atherosclerosis

Despite prevention measures, early identification of atherosclerosis, the common pathophysiological process that causes underlying cardiovascular disease, is difficult to achieve, with overt coronary artery disease or myocardial infarction considered the most commonly initial clinical manifestation. A novel approach to the detection, prevention, and treatment of atherosclerosis is presented by nanoparticles. Concerning the diagnosis of atherosclerosis, new multifunctional nanoparticles with integrated diagnostic and therapeutic capabilities are promising (103). Metal nanoparticles have been used in studies for the specific imaging or treatment of atherosclerosis. CD163 refers to a membrane receptor from the macrophage lineage. Studies on atherosclerosis have revealed higher levels of CD163 expression in inflammation-related locations, indicating the presence of intraplaque hemorrhagic sites or asymptomatic plaques. Consequently, the imaging of CD163-expressing macrophages wokrs as a novel method for the detection of atherosclerotic plaques. For the precise detection of CD163 by MRI, Carlos et al. created a targeted probe based on gold-coated iron oxide nanoparticles vectorized with an anti-CD163 antibody (104).

In recent years, inflammatory biomarkers have been demonstrated to act as an effective signal for early diagnosis of atherosclerosis (105). Integrins, macrophage scavenger receptors, VSMCs, and VCAM-1 on endothelial cells are among the most frequently detected biological targets. The VCAM-1 expression is induced early in human atheroma and represents a key element of the inflammation generated during atherosclerosis, contributing to monocyte and lymphocyte recruitment from adventitial vessels and the arterial lumen. The strict temporal and spatial regulation of endothelial adhesion molecules and their critical function in atherosclerosis make them ideal targets for diagnosis (106). Besides, there are many microstructures in late-stage atherosclerotic plaques, such as neovascularization, micro-calcification, and cholesterol crystals, which have become important foci for targeted nanomedicine delivery (107).

It has been demonstrated that atherosclerotic plaque locations display high permeability and retention effect similar to solid tumors. AuNPs drive macrophage depletion in the photothermal therapy (PTT) by converting light energy to heat energy under atherosclerotic conditions. PTT actively makes use of inorganic nanoparticles and spatial near-infrared laser wavelengths to cause intracellular hyperthermia. It is shown that gold nanorods and silicon-gold hybrid nanoparticles function in concert with PTT after the total atherosclerotic volume is reduced (106).

###### 2.3.2.2.2. Nanotherapy for myocardial infarction

In the last few years, nanoparticles such as GNP, carbon dots, and graphene have continued to be key protagonists of different types of sensors (e.g., electrogenerated chemiluminescence sensor) to enable multiple and ultrasensitive detections of relevant cardiac biomarkers such as cTnI, cTnT, myoglobin, and glutathione (108, 109).

During the myocardial damage process, the cardiac troponin I (cTnI) is released into the bloodstream. Early detection of cTnI in the serum of patients with a higher risk of MI can decrease the risk of death. For instance, Guo et al. developed a facile and rapid solution-phase method to detect human cTnI using anti-human cTnI-labeled GNR-based biosensors (110).

Reactive oxygen species are eventually produced due to myocardial ischemia and hypoxia, which cause the pathophysiological circumstances of MI. Decreased glutathione expression is another effect of the steric overproduction of ROS. Therefore, measuring glutathione levels can help diagnose MI and ischemic heart disease. In this context, important to the pathophysiology of MI, glutathione levels are detectable using carbon-dot-based ultrasensitive biosensors. The addition of graphene oxide, mesoporous silica, and silver nanoparticles increased the sensitivity of the carbon dot-based biosensors. Carbon dots and mesoporous silica work well together as supersensing components in biosensors based on conjugated nanomaterials. Therefore, by supplying the required information for future therapy, this notion can aid in the early diagnosis of MI (109).

During the MI, the heart sustains irreparable damage (111). Factors such as the start of a pro-inflammatory cytokine cascade and the production of ROS and RNS contribute to the pathophysiology of TNF-mediated MI (112, 113). Therefore, a key technique for treating MI is effectively decreasing TNF during the inflammatory phase (114, 115). We notice that some novel payloads in NPs-based therapy for MI have been studied. For example, deoxyribozyme-AuNP can silence tumor necrosis factor-α (TNF-α) (116). As a potential treatment for myocardial infarction, they employed gold nanoparticles (AuNPs) functionalized with deoxyribozyme (DNAzyme) to catalytically quiet tumor necrosis factor (TNF) *in vivo*. They showed a 50% knockdown of TNF-α in primary macrophages, which was not possible with Lipofectamine-based techniques. TNF-α knockdown efficiencies of 50% were achieved following local injection of DNAzyme attached to gold particles (AuNPs) in the rat myocardium, which had strong anti-inflammatory effects and improved immediate cardiac function after MI. These findings are the first evidence to demonstrate the viability of DNAzyme AuNPs conjugates for gene regulation and delivery *in vivo*. This is important because TNF-α has been associated with numerous inflammatory-mediated illnesses and is a multibillion-dollar treatment target, highlighting the potential effects of DNAzyme-conjugated AuNPs (116).

## 3. Biomimetic nanocarriers

### 3.1. Development of biomimetic nanocarriers

Nano-drug carriers can encapsulate water-soluble or poorly soluble drugs. They can also simultaneously encapsulate multiple drugs to co-deliver them. They can achieve sustainability, controllability, intelligence, responsiveness, and so forth. However, it is difficult for them to imitate the natural biological structure effectively (117). Non-biological exogenous factors are inevitably brought into the body when the drug is delivered, decreasing efficacy. On the contrary, biomolecules, such as blood cells, can move freely as the blood is circulated without being opsonized or cleared. Their unique function is to protect the immune system from invading normal cells without being detected (118).

Similarly, drugs are in an environment similar to biological materials before reaching the target site. Therefore, some scholars seek to develop new drug delivery systems of drug carriers according to their biological properties and functions using biomimetic technology. In recent years, there has been continuous research on efficient and specific drug delivery using biological materials such as cell membranes, proteins, viral capsids, and liposomes as carriers (119) (Figure 1).

### 3.2. Classification of biomimetic nanocarriers

#### 3.2.1. Related delivery systems for proteins, viral capsids, and liposomes

The biomimetic synthesis method using proteins as a template is a new method for nanoparticle preparation. Amino acid residues in protein molecules can interact or coordinate electrostatically with metal ions to form nucleation centers. In the cavity, the nucleation and growth of inorganic nanocrystals are induced by reduction reactions, precipitation reactions, and so forth, thereby forming protein template-based nanoparticles. The method is simple to prepare, with adjustable particle size and good biocompatibility (120).

Some researchers have developed virus-like particles (VLPs) and virions. VLPs are a class of self-assembled particles that mimic the structure of viral capsids (121). The particles’ empty architecture can be filled with a variety of materials including proteins (122), nucleic acid (123), drugs (124), and other nanoparticles (125), and they no longer contain viral genetic material. VLPs and virions can promote cellular uptake and avoid lysosomal phagocytosis, thus achieving targeted delivery. However, biosafety remains a controversial issue (126).

As materials science and nanotechnology keep maturing, many nano-formulations, such as liposomes, micelles, and nanoparticles (NPs), have been developed and used in clinical practice (127). Among them, liposomes are considered an effective drug delivery system (DDS), and over ten liposome drugs have been approved to go on the market (128). Liposomes have many advantages in drug delivery, such as good biocompatibility, biodegradability, tunable size, and surface modification (129). They are often used as embedded hydrophilic or hydrophobic reagents, improving the circulation of drugs *in vivo*. More importantly, its chemical composition (phospholipids) and lipid bilayer structure resemble biological membranes and are highly compatible with the biological environment. In addition, modifying targeting groups can enhance drug delivery efficiency (130).

Biomimicry, such as liposomes, has been used to construct biofilm models to study the biological functions of living cells (131, 132). However, due to its relatively simple structure, it is difficult to reproduce cell membranes’ complexity accurately.

#### 3.2.2. Cell or cell membrane-based drug delivery systems

In recent years, people have tried to use natural cells or cell-derived vesicles as drug carriers, such as whole cells, extracellular vesicles (EVs), and membrane-encapsulated nanoparticles (133–135). Because they resemble cell membrane structures, they are recognized by the body as “self” components, showing better biocompatibility and lower toxicity. Simple to prepare, this drug delivery system can reduce the loss of membrane proteins and endow the carrier with multiple biological functions and targeting specificity without further modifications. For example, red blood cells (RBCs) are used as carriers to encapsulate small molecule drugs, nucleic acids, proteins, nanoparticles, and so forth in treating systemic diseases due to their long lifespan and good biocompatibility (136, 137). RBCs, stem cells, macrophages, neutrophils, T cells, and NK cells are just a few of the cells that have been employed for drug loading and delivery. In contrast to cell membrane-camouflaged DDSs, recent research suggests that cell-based DDSs can retain all the biological characteristics and functions of native cells, including the long lifespan of erythrocytes (red blood cells, RBCs) in circulation, the inflammatory homing ability of stem cells, macrophages, and neutrophils, and the tumor recognition and killing abilities of T cells and natural killer cells. Consequently, they are able to get beyond the drawbacks of artificial NP carriers (138).

##### 3.2.2.1. Whole cells as drug carriers

The physiological functions of various cells in the human body vary, such as long blood circulation, specific site migration, and crossing physiological barriers. We can select specific cells to deliver drugs using their retained cellular structures and functions. Recently, the design of utilizing whole cells (erythrocytes, stem cells, immune cells, etc.) as drug carriers has become a hotspot (138).

Erythrocytes are the most employed carrier of whole cells. Compared with synthetic drug delivery systems, erythrocytes display the advantages of better biocompatibility and biodegradability with no immunogenicity. Their natural surface protects the loaded drug from being inactivated to prolong circulation time and enhance controllability, which makes the systemic drug delivery systems valuable (139). Various methods have been developed to encapsulate drugs into red blood cells to modify drugs on the surface of red blood cells chemically or physically (140).

Inflammation sites can recruit immune cells (macrophages, lymphocytes, neutrophils, etc.) (141, 142). RNA-loaded liposomes, magnetic nanoparticles, gold nanoshells, and imaging agent-loaded nanoparticles are easily engulfed by monocytes and neutrophils during circulation in the body, and immune cells can selectively cross the blood-brain barrier to allow therapeutic drugs to reach the brain (143–145).

##### 3.2.2.2. Cell membrane-encapsulated nanoparticles as drug carriers

Membrane-based vesicles are natural drug carriers that have been used in various systems. However, due to their preparation method and liposome structure, it is challenging to load hydrophobic agents, co-deliver drugs of different properties, and produce well-controlled release (146). It is well known that polymeric or inorganic nanoparticles can fulfill the above functions, but due to their heterogeneity, they are rapidly cleared by the body. Similar to lipid-encapsulated hybrid nanoparticles, a series of cell membrane-encapsulated nanoparticles have been developed with the advantage of being multi-functional (147).

Since red blood cell membrane (RBC) encapsulation technology was developed in 2011 by Zhang et al. it has been applied to multiple systems for varying purposes (148, 149). The technology adopts a three-step method to prepare novel erythrocyte membrane-encapsulated nanoparticles. First, red blood cells are separated from the whole blood by centrifugation, then hemoglobin is removed with a hypotonic solution. Next, through extrusion molding of a polycarbonate porous membrane (100 to 400 nm), RBCm vesicles with a size of about 100 nm are obtained. The final step is to co-extrude the RBCm vesicles and nanoparticles multiple times to fuse. A lipid bilayer is observed outside the nucleus of the nanoparticles, with a surface potential close to that of RBCm vesicles. Both results confirm the successful encapsulation of the red blood cell membrane.

It is worth noting that the erythrocyte membrane-encapsulated nanoparticles synthesized by this method maintain the original membrane orientation. That is to say, the face is facing outward, and most of the protein content and glycogen density on the membrane surface are similar to those of erythrocyte, capable of stabilizing the physicochemical stability of nanoparticles, thereby prolonging the *in vivo* circulation time of erythrocyte membrane-coated nanoparticles, which is much longer than that of liposome-based nanoparticles (150). The chemotherapeutic drug doxorubicin DOX nanoparticles can be wrapped around the erythrocyte membrane through chemical coupling or physical encapsulation (151).

Zhang et al. extracted neutrophils (NEs) from mice and loaded paclitaxel PTX liposomes with neutrophil membranes as templates. NE can naturally cross the blood-brain barrier/blood-brain tumor barrier (BBB/BBTB) and glioma sites. Surgical resection of gliomas can release local inflammatory factors, activate NE to migrate to the inflammatory site in the brain, and “amplify the signal,” thereby improving the targeting of brain tumors and significantly inhibiting the recurrence of malignant gliomas after surgery (152, 153).

### 3.3. Applications

#### 3.3.1. Biomimetic nanomaterials and other inflammation-related diseases

Thamphiwatana et al. (154) obtained the purified macrophage membranes by extracting J774 mouse macrophages through hypotonic lysis, mechanical destruction, and differential centrifugation. They used ultrasound to transform the macrophage membranes into membrane vesicles and mixed them with PLGA. It was discovered that the nanoparticles reduced the inflammation caused by LPS-induced sepsis in mice and increased their chance of survival under the condition.

Considering the critical role of neutrophils in sepsis-induced liver injury, Xiao et al. explored neutrophil cell membrane-mimicking nanomaterials (NM) as biomimetic nanomaterials for treating the sepsis-related liver injury. In a mouse model with sepsis, NM administration showed good biocompatibility and significantly reduced plasma levels of inflammatory cytokines and biomarkers of liver injury, including aspartate aminotransferase, alanine aminotransferase, and direct bilirubin. Therefore, it provides a promising therapeutic strategy for treating acute liver injury caused by sepsis (155).

Organ transplantation is often the last treatment for end-stage patients with organ diseases. Transplant rejection and inflammatory injury (such as ischemia-reperfusion) account for most failures in organ transplantation (156). Transplant tolerance is present between donors and donors. Progress in similar research has also been made in liver and skin transplantation (157, 158).

Arthritis, a chronic inflammation-related disease, is a common cause of clinical teratogenicity and disability (159). Although progress has been made in studies concerning the clinical application of anti-cytokine biologics, the therapeutic effect on arthritis remains unsatisfactory, especially with the low response rate of rheumatoid arthritis. Biomimetic nanomaterials are also used to treat arthritis (160, 161).

In addition to the diseases mentioned above, many studies have shown the applications of biomimetic nanomaterials in other inflammation-related diseases, such as inflammatory bowel disease, trauma, etc (7).

As a public health disease, COVID-19 is still raging around the world. Pathophysiological studies have shown that virus-induced cytokine release syndrome is the leading cause of death (162, 163). Knowing that the infectivity of COVID-19 depends on its interaction with known or unidentified protein receptors on target cells, Zhang et al. created a cellular nanosponge that primarily impacts host cells as opposed to pathogens (164).

#### 3.3.2. Biomimetic nanomaterials and cardiovascular disease

Cardiovascular disease is a severe and threatening disease as one of the leading causes of death worldwide (165). Common cardiovascular diseases, such as atherosclerosis and valvular heart disease, are chronic systemic inflammatory diseases caused by obesity, autoimmune diseases, and infectious diseases, leading to cardiovascular disease occurrence and exacerbation (166). Biomimetic nanomaterials are also currently used to prevent and treat cardiovascular diseases (167). For instance, Hu et al. obtained a new biomimetic valve by cross-linking erythrocyte membrane-coated drug nanoparticles and an artificial heart valve, which reduces the toxic effect of glutaraldehyde on the heart valve but also considerably improves the tissue of the artificial heart valve (168).

##### 3.3.2.1. Applications in the diagnosis and treatment of atherosclerosis

Atherosclerosis is one of the pathological bases of cardiovascular disease. It is mainly a progressive inflammatory disease involving lipid deposition and massive accumulation of immune cells, eventually forming a fibrous cap on the arterial wall. Among them, dyslipidemia is the most important influencing factor of atherosclerosis. A recent study designed dual-targeted biomimetic nanocomposite particles with a core-shell structure. The research results showed that through a positive feedback loop, the novel multifunctional biomimetic nanoparticles could dynamically enhance the targeting of plaques while achieving the dual effects of inhibiting cholesterol deposition and enhancing efflux. It provides a new treatment to reduce plaque formation and alleviate atherosclerosis (169). Gao et al. and Wang et al. prepared reactive oxygen species responsive nanoparticles (ROS-responsive NPs). They then coated them with the extracted macrophage membrane to obtain the macrophage membrane-coated ROS-responsive nanoparticles. Nanoparticles effectively avoid being cleared by the body’s reticuloendothelial cells, help to target the inflammatory site, realize the sustained release of drugs in the inflammatory site, and reduce the production of reactive oxygen species and the risk of atherosclerotic disease in mice (170, 171). In the sclerosis model, this biomimetic nanomaterial showed a superior therapeutic effect. Based on a similar method, Boada et al. loaded rapamycin on nanoparticles and then coated the nanoparticles with a macrophage membrane. The biomimetic nanomaterials improved the histocompatibility and sustained the release efficiency of the drug. In the mouse model with high-fat diet-induced vasculitis, this newly drug-loaded biomimetic nanoparticle better relieved the vascular inflammation and organ damage to mice (172). Song et al. used PLGA nanoparticles to encapsulate rapamycin and the outer membrane of red blood cells to treat atherosclerosis. They found that red blood cell membranes could reduce the phagocytosis of nanoparticles by macrophages in the blood circulation and enhance their phagocytosis. Aggregation in plaques to achieve sustained release of rapamycin reduces atherosclerotic plaques. Platelet membranes have been used in some studies to modify PLGA rapamycin nanoparticles and obtained similar effects (173).

In addition to reducing the formation of atherosclerotic plaques, the biomimetic nano-delivery system can also be used as an imaging tool to assess the progression of atherosclerosis (174). Zhang et al. took advantage of the natural feature of platelets involved in the disease to extract platelet membranes. Construction of platelet membrane-encapsulated PLGA Nano-Bionic Particles (PNPs). The contrast agent gadolinium is modified on the surface of PNPs to obtain the targeted modal contrast agent GdPNP. MRI analysis showed strong positive contrast 1 h after administration, indicating that Gd-PNP can target the plaques accumulating in the aortic arch in a short time. The formation site enables MRI imaging at the plaque to track the progression of atherosclerosis (175).

##### 3.3.2.2. Role in the repair of cardiac injury

Cardiac damage is mainly due to ischemic necrosis of myocardial cells caused by long-term stenosis or the occlusion of coronary arteries. At the same time, the myocardial ischemia site has vascular endothelial cell damage, collagen exposure, high vascular permeability, and many inflammatory cells, including macrophages. The current clinical drugs for the treatment of myocardial ischemia mainly rely on growth factors, cytokines, and small molecular compounds to improve symptoms and delay cardiac injury. As cell membranes or membrane-like structures have evolved, biomimetic nano-drug delivery systems are expected to achieve efficient “active targeting” of nanoparticles. A platelet-inspired nanocell (PINC) that incorporates prostaglandin E2 (PGE2)-modified platelet membrane and cardiac stromal cell-secreted factors to target the heart after ischemia-reperfusion injury (I/RI) is intr.oduced. The dual roles of targeting and repairing the damaged heart can thus be achieved (176). Studies have confirmed that microvesicles such as exosomes secreted from the iPSC-derived cardiomyocytes exert protective effects by transferring the endogenous molecules to salvage the injured neighboring cells by regulating apoptosis, inflammation, fibrosis, and angiogenesis (177). However, how to efficiently deliver it to the heart remains to be explored. Cardiomyocyte-specific peptides are linked to amp2b-positive exosomal membrane structures, thereby enhancing exosome endocytosis in cardiomyocytes (178). Alternatively, the peptides targeting the heart can be conjugated to exosomes by the linker to make exosomes target the damaged heart more effectively, thereby reducing myocardial fibrosis in the damaged heart. Some of these effects are mediated by the transfer of microRNA (miRNA) to the heart (179).

Huang et al. constructed a long-circulation sustained-release H_2_S system to explore how it prevented myocardial ischemia-reperfusion (I/R) injury. Red blood cell (RBC) membrane diallyl trisulfide (DATS) supported mesoporous iron oxide nanoparticles (MIONs) (RBC-DATs-MIONs) were highly biocompatible. They prolonged the circulation time and controlled the release of H_2_S in the plasma and myocardium. They showed excellent therapeutic efficacy in an *in vitro* hypoxia/reoxygenation and *in vivo* myocardial I/R model involving multiple mechanisms. For instance, the anti-apoptotic, anti-injury, and antioxidant activity (180).

Su et al. intr.oduced a platelet-excited nanocell (PINC) that incorporates prostaglandin E2 (PGE2) -modified platelet membrane and cardiac stromal cell-secreted factors to target the heart after the I/R injury. In a mouse model of the myocardial I/R injury, intravenous PINCs enhanced cardiac function and attenuated cardiac remodeling. Meanwhile, they improved cardiomyocyte circulation, activated endogenous stem/progenitor cells, and promoted angiogenesis (176).

Lin et al. prepared a biointelligent nanoparticle (MTSNP) with microenvironment targeting and adaptability to repair the ischemic tissue. During the acute phase of ischemia, melatonin is rapidly released from MTSNP. The process scavenged reactive oxygen species, activated melatonin receptor I on MT, and prevented cytochrome C release, activating apoptosis. In the chronic phase, circular DNA could sense the hypoxia and secreted VEGF to revascularize as a response. Importantly, circular DNA might adversely affect receiving feedback from the revascularization and shut down VEGF secretion. The therapeutic potential of MTSNP in myocardial ischemia was verified by various methods (181).

##### 3.3.2.3. Application in other cardiovascular diseases

Thrombosis is involved in the pathophysiological process of diseases, such as atherosclerosis and MI. Nie et al. used platelet nanoparticles extracted from platelets to carry thrombolytic drugs in the targeted thrombolytic therapy (182). Clinically, in-stent restenosis occurs when the platelet aggregates, excessive smooth muscle cell proliferates, and inflammatory cell aggregates. The current research mainly focuses on endothelialization after stent placement. The research is based on constructing liposomes or PLGA nanoparticles to transport proteins or genes such as vascular endothelial growth factor and angiopoietin (183).

## 4. Conclusion

In conclusion, biomimetic nanomaterials and biomimetic nanodelivery systems are anticipated to become potentially beneficial technologies for disease diagnosis and therapy, enabling nanomedicine to represent significant potential value for medical applications in the diagnosis and treatment of the cardiovascular system. However, its applications still face considerable challenges: biostability, biodistribution, biocompatibility, and biosafety of nanoparticles, for instance. As a result, nanoparticles that are highly selective, toxic-free, and more applicable in therapeutic settings are required. Further optimization is necessary to target biomimetic nanoparticles, high ligand affinities, circulation *in vivo*, and cytotoxicity. Biomimetic nanomedicine research is still in the early stage until biomimetic nanoparticles that target inflammation are intr.oduced into clinical settings.

## Author contributions

All authors participated in the creation of this manuscript, contributed to the article, and approved the submitted version.
